# The leaky pipeline of publications and knowledge generation in medical education

**DOI:** 10.1007/s40037-022-00700-4

**Published:** 2022-03-03

**Authors:** Rashmi A. Kusurkar

**Affiliations:** 1 Amsterdam UMC, Research in Education, Faculty of Medicine, Vrije Universiteit, Amsterdam, The Netherlands; 2 LEARN! research institute for learning and education. Faculty of Psychology and Education, VU University Amsterdam, Amsterdam, The Netherlands

My journey in medical education is a global one. I relocated as a doctor, teacher and researcher from the Global South (India) to the Global North (the Netherlands). As I reminisce about my ambitious, motivated self in India with competence in research, but low confidence in scientific writing and publishing, I realize that I simply did not have the training and resources to complete the trajectory from conducting innovative research to crafting a publication-worthy academic paper. By hindsight, my research was publication-worthy, my writing was not. Moving to the Netherlands gave my career a new boost. I was granted access to resources at a premier Dutch institute, University Medical Center Utrecht, and could benefit from the guidance of world-renowned expert Professor Olle ten Cate. By virtue of my new location and my new mentor, I became part of a network where the dominant knowledge conversations in the field were happening. I became privy to those conversations and viewpoints. I was able to identify gaps in the literature relatively easily through reading and talking to people about research and was successful in setting up my own research program with innovative research themes.

After my PhD, as I gathered experience as a reviewer, I came across interesting manuscripts in medical education written by authors from the Global South that were rejected for reasons such as: ‘the gap in the literature is not clear’, ‘poor quality of scientific writing’, ‘poor English language skills’, ‘this has already been done in the Western context’, ‘this topic is not important enough in the current conversations in the field’, and so on. It broke my heart to see high-quality original work rejected for these reasons, knowing it had been conducted under serious resource constraints by researchers who were not familiar with the publication culture in the Global North. I identified with each of those authors, who I could imagine had overcome major hurdles in order to put pen to paper and muster enough confidence to submit their work. When I was appointed as an associate editor on multiple medical education journals, I made a pact with myself that I would drive the cause to help authors from the Global South publish in the major journals. I would do this by mentoring some authors on a one-on-one basis, while simultaneously starting conversations about this topic with editors. This commentary on the leaky pipeline in publications in medical education is an attempt to drive this conversation.

## The leaky pipeline

**Fig. 1:**
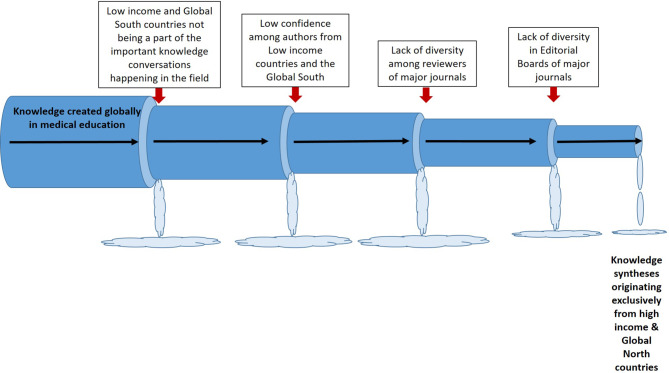
The leaky pipeline of publications in medical education

The leaky pipeline metaphor has been used to illustrate the lack of representation of women in academia [1] and in the STEM (science, technology, engineering, and mathematics) disciplines [2], and the lack of people of colour in academia as well as in medicine [3]. The leaky pipeline metaphor fits medical education publication practices beautifully. Knowledge on medical education is generated all over the globe, but due to systematic factors in the field only certain types of knowledge are published and acknowledged as ‘knowledge’ that adds to the field. In Fig. 1, I illustrate the different points in the knowledge pipeline where knowledge from the Global South is lost and never reaches or is never incorporated into the Global North medical education research reservoir.

Buffone et al. conducted an investigation into publications in seven major medical education journals over a 10-year period and reported that publications with lead authors from the Global South (Asia, Africa and South America) were less than 21%, even though these continents represent around 85% of the world population [4]. Maggio et al. conducted an analysis of knowledge syntheses in medical education, uncovering who drives the knowledge conversations in medical education [5]. They reported that only 6% of knowledge synthesis in health professions education included authors from low or middle income countries. Among the remaining 94% of authors, majority of articles were written by authors from highly ranked institutions from high income countries. Thus, the power structure of knowledge is extremely skewed to certain institutions from high income countries, and especially from North America, (which represents only around 5% of the world population).

In their recent investigation of the editorial boards of ten well-established medical education journals, Yip and Rashid brought to light a well-known picture that was never before supported by hard data [6]. On a scale of 0–10, six journals scored poorly (≤ 5) on a composite score comprising gender-related, geographic and income level-related diversity, within which the scores of most of them were accounted for by gender diversity rather than geographic and income level-related diversity. Unfortunately, ‘Perspectives on Medical Education’ scored only 5 on this scale and ‘Academic Medicine’ was reported to have a fully North American Editorial Board.

Thus, it begins with the Global South being excluded from conversations in the medical education field. An article published by Lancet Global Health on how the Global South is not part of the storytelling in the medical field is evidence of this phenomenon [7]. Global South representatives are not at the table to discuss the medical education research agenda. I am reminded of the saying, ‘If you are not at the table, you will end up on the menu’. On top of that, unconventional competences (such as in writing powerful personal narratives, inclusion of traditional wisdom in science, etc.) and the new domains of knowledge generated in the Global South are not accepted as legitimate [8]. Thus, work from the Global South is at the mercy of authors, reviewers and editors from the Global North, who may not understand the grounded reality of medical education in the Global South [6, 8].

Lack of resources or access to literature and training in Western scientific writing, which is the dominant accepted practice, can seriously hamper the confidence and perceived competence of Global South authors. Much of the work is not even submitted for publication in major journals. If some authors do gather the guts to submit their work, which seems to be happening in the last few years, there is still only a miniscule chance that this work will be accepted for publication [8].

If we put the lack in diversity of authors successful in publishing in major health professions education journals together with the lack of diversity of editorial boards, it is reasonable to conclude that the extremely skewed knowledge syntheses is informally and implicitly dominated by a handful of powerful Global North (associate) editors. They set standards for quality which is not open for the majority to debate [8]. Our leaky pipeline needs urgent investigation and the data already exist. A program of research needs to be set up globally for investigating the grounded reality. This should include: a) analysis of diversity among reviewers, b) analysis of reviewers’ recommendations on acceptance/rejection of papers written by authors from low and middle income countries and geographically diverse regions, and c) how these decisions are accepted/rejected by editorial boards to determine final acceptance/rejection decisions.

## How can we address this problem?

Addressing this problem needs a combination of several strategies such as:

*Related to publications practices*—Naidu recommends a three-pronged strategy to address the issue of publication bias and calls it ‘*decolonial praxis*’: a) Changing the actors—By providing marginalized authors the opportunities to publish in major journals, b) Shifting the power in research—By funding context-relevant unconventional research, which is not embedded in Western frameworks, and c) Dismantling the predominant writing structures and fixed notions of the quality of science/research [9]. I, for one, would welcome such initiatives to tackle this problem and to bring equity in medical education publication practices and knowledge generation, the suggestion of which comes from an author from the Global South.

*Related to journal editorial boards*—Journal Editors must take up diversity targets as some of their key performance indicators. This includes geographic as well as income-level related diversity not only among the authors whose work they publish but also in their Editorial Boards. Every journal should publish a report on diversity in their publications annually, in the interest of transparency in publication practices.

*Related to specific initiatives for enhance research and publication from the Global South*—International initiatives such as the International Network for Advancing Science and Policy (INASP) can be considered as best practices to learn from [10]. INASP strives to produce an equitable knowledge ecosystem in the world through including every voice and fostering every talent. They provide support and guidance at three levels: individual, organizational and systemic. Individual support comprises inclusive production, communication, appraisal, and use of knowledge. Organizational support comprises developing inclusive environments for critical thinking, learning and knowledge production. Systemic support comprises coproduction, and using relevant and quality evidence in teaching and learning [10]. We need more initiatives such as INASP to create a critical mass of research and publications from the Global South.

This is a call to join forces to fix our leaks!

## References

[1] Wickware P (1997). Along the leaky pipeline. Nature.

[2] Blickenstaff JC (2005). Women and science careers: leaky pipeline or gender filter?. Gend Educ.

[3] Barr DA, Gonzalez ME, Wanat SF (2008). The leaky pipeline: factors associated with early decline in interest in premedical studies among underrepresented minority undergraduate students. Acad Med.

[4] Buffone B, Djuana I, Yang K, Wilby KJ, Hajj MSE, Wilbur K (2020). Diversity in health professional education scholarship: a document analysis of international author representation in leading journals. BMJ Open.

[5] Maggio LA, Ninkov A, Costello JA, Driessen EW, Artino AR (2021). Knowledge syntheses in medical education: meta-research examining author gender, geographic location, and institutional affiliation. PLoS ONE.

[6] Yip SWL, Rashid MA (2021). Editorial diversity in medical education journals. Clin Teach.

[7] The Lancet Global Health (2021). Global health 2021: Who tells the story?. Lancet Glob Health.

[8] Naidu T (2021). Says who? Northern ventriloquism, or epistemic disobedience in global health scholarship. Lancet Glob Health.

[9] Naidu T (2021). Modern medicine is a colonial artifact: introducing decoloniality to medical education research. Acad Med.

[10] Unleash the talent: towards an equitable ecosystem. INASP strategy 2020–2025.. https://www.inasp.info/sites/default/files/2020-05/INASP-2020-Strategy-DIGITAL-Compressed.pdf. Accessed 11 Jan 2021.

